# Effect of Temperature on Corrosion of HSLA Steels with Different Cr Contents in a Water-Saturated Supercritical CO_2_ Environment

**DOI:** 10.3390/ma18225243

**Published:** 2025-11-20

**Authors:** Qilin Ma, Shilin Liu, Yi Ren, Leng Peng, Ba Li, Chengjia Shang, Shujun Jia

**Affiliations:** 1 Central Iron and Steel Research Institute Co., Ltd., Beijing 100081, China; maqilin0812@163.com (Q.M.); lengpeng4782@126.com (L.P.); balicugb@sina.com (B.L.); 2 Collaborative Innovation Center for Steel Commonality, University of Science and Technology Beijing, Beijing 100083, China; cjshang@ustb.edu.cn; 3 Construction Project Management Branch of China National Petroleum Pipeline Network Group Co., Ltd., Langfang 065000, China; liusl01@pipechina.com.cn (S.L.); renyi04@pipechina.com.cn (Y.R.); 4 School of Metallurgy and Energy, Kunming University of Science and Technology, Kunming 650093, China

**Keywords:** supercritical CO_2_, corrosion behavior, Cr content, temperature effect, thermodynamic and kinetic analysis

## Abstract

This study investigates the effects of chromium (0.4~1.2) Cr content and temperature (35–80 °C) on the corrosion behavior and mechanisms of steels in a water-saturated supercritical CO_2_ (S-CO_2_) environment, aiming to provide theoretical foundations for material selection and corrosion management in S-CO_2_ pipeline systems. Results indicate that increasing Cr content promotes the formation of granular bainite as the dominant microstructure, accompanied by refined martensite–austenite (MA) constituents with increased population and reduced dimensions, leading to enhanced strength at the expense of toughness. In the S-CO_2_/H_2_O environment, Cr reacts with CO_2_ to form a dense Cr_2_O_3_ layer, significantly suppressing the corrosion rate. Temperature critically governs corrosion kinetics: at 35 °C, where S-CO_2_ exhibits maximum density and CO_2_ solubility in water peaks, electrochemical corrosion dominates, resulting in the highest corrosion rate. As temperature rises, the corrosion mechanism transitions to chemical corrosion, while accelerated formation of protective corrosion product films further reduces corrosion rates. Mechanistic analysis reveals that uniform corrosion arises from carbonic acid generated by water dissolution in S-CO_2_, whereas localized corrosion intensifies upon direct contact between precipitated aqueous phases and the steel surface. These findings offer critical theoretical foundations for optimizing material design, operational parameters, and corrosion mitigation strategies in S-CO_2_ transportation infrastructure.

## 1. Introduction

With the growing issues of the greenhouse effect and ocean acidification, environmental concerns regarding carbon dioxide (CO_2_) emissions have become increasingly urgent [1]. Since the Global Climate Conference, Carbon Capture, Utilization, and Storage (CCUS) technologies have rapidly advanced. These technologies aim to curb the continuous rise in atmospheric CO_2_—thereby mitigating its impact on ecosystems while also generating economic benefits—by capturing, transporting, and ultimately utilizing CO_2_ for applications such as enhanced oil recovery and extraction [2,3]. CCUS processes are generally divided into four segments: carbon capture, transportation, utilization, and injection. Although carbon capture and utilization have received significant attention and are relatively mature, the transportation phase, a critical component of CCUS, has been comparatively understudied. Among various transportation methods, pipeline transport is the most common due to its economic viability, safety, and efficiency [4]. Moreover, to accommodate the increasing CO_2_ pressure and avoid two-phase flow issues, CO_2_ is typically compressed into a supercritical state during pipeline transport [5,6].

However, supercritical CO_2_ poses significant corrosion challenges during pipeline transport, with its corrosiveness increasing as water content rises in the system [7]. In addition, environmental factors such as temperature and pressure, along with the presence of impurity gases, influence the corrosion process. Specifically, the combined action of impurity gases and CO_2_ can generate more aggressive corrosive media, leading to uniform corrosion, pitting, stress corrosion cracking, and other degradation forms in pipeline materials. Although extensive studies have examined the effects of impurity gases on supercritical CO_2_ corrosion [8,9,10,11]—with Li K et al. [12] reviewing the influence of various impurities on corrosion rates, products, and progression—this study shifts focus. The effects of Cr content and temperature on corrosion behavior in water-saturated supercritical CO_2_ (in the absence of impurities) are systematically examined. Prior research indicates that Cr can mitigate CO_2_ corrosion; however, the coupling effects of Cr content and temperature remain insufficiently explored. For example, Han et al. [13] found that increasing Cr content tends to reduce the corrosion rate of supercritical CO_2_, while elevated temperatures exacerbate corrosion in Fe–Cr alloys. Although these findings offer valuable insights into developing steels for supercritical CO_2_ transport, the composition systems tested are not ideally suited for conventional transport and are cost-prohibitive. Li et al. [14] also investigated the corrosion behavior of steels with varying Cr contents in S-CO_2_ environments containing impurities at 10 MPa and 45 °C, providing further inspiration despite notable differences from actual operating conditions. Therefore, this study aims to elucidate the mechanism by which Cr influences corrosion in a water-saturated supercritical CO_2_ environment free of additional impurities, thereby contributing to enhanced pipeline material quality and improved safety standards.

Theoretically, completely dry supercritical CO_2_ is non-corrosive; however, its corrosivity increases with rising water content. Kowta R et al. [15] modeled the maximum solubility of water in supercritical CO_2_ without considering gaseous impurities—a finding that is essential for developing future pipeline gas standards. Hua et al. [16] investigated the corrosivity of water-saturated supercritical CO_2_ at 35 °C and 50 °C, noting that corrosivity appears to decrease with increasing temperature. They attributed this trend to the enhanced formation of protective corrosion product films, which impede further substrate degradation. However, this explanation alone is insufficient, as the study only examined two temperature points, and research at temperatures above 50 °C remains limited. In contrast, Sui et al. [17] studied water-saturated supercritical CO_2_ containing H_2_S impurities and observed that the corrosion rate initially increased with temperature before subsequently decreasing, with the highest rate occurring at 35 °C. While these findings advance the understanding of corrosion in supercritical CO_2_ environments, the direct link between changes in corrosion rate and supercritical CO_2_ density is less clearly established. Moreover, the interplay between the protective characteristics of corrosion products and the overall corrosive strength requires further comprehensive analysis. Future investigations should address these complexities to provide a more in-depth understanding of the corrosion mechanisms in supercritical CO_2_ systems.

The focus of this work is to elucidate the effects of temperature and Cr content on the corrosion behavior of water-saturated supercritical CO_2_. Three sets of specimens with varying Cr contents (0.38, 0.75, and 1.17 wt.%) were subjected to corrosion tests in water-saturated supercritical CO_2_ environments at different temperatures (35 °C, 50 °C, and 80 °C) under an 8 MPa pressure. The basic microstructure and mechanical properties of the experimental steels were characterized through metallographic, scanning electron microscopy, and mechanical property tests. Corrosion rates were determined by weight-loss measurements, and the cross-sectional morphology and composition of the corrosion products were analyzed using SEM, EDS, and XRD to elucidate the role of Cr in forming protective rust layers. Additionally, thermodynamic modeling was employed to calculate the solubility of water in supercritical CO_2_ and the solubility of CO_2_ in the aqueous phase. Based on these results, a corrosion model applicable to water-saturated supercritical CO_2_ was developed to explain the mechanisms by which temperature influences corrosion behavior. This study provides valuable insights for enhancing pipeline safety and extending service life in CO_2_ transport applications.

## 2. Materials and Methods

**Figure 1:**
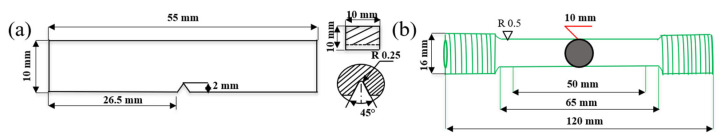
Shape and dimensions of impact and tensile test specimens. (Unit: mm). (**a**): Low temperature impact specimen, (**b**): Round bar tensile specimen [23].

The experimental materials were selected from three kinds of test steels rolled by ANSTEEL, and their chemical compositions are detailed in Table 1. The experimental materials were first prepared into ingots by vacuum melting and forging, and then heated to 1180 °C and held for 1.5 h to eliminate forging defects and promote the re-solid solution of microalloying elements into austenite [18,19]. Subsequently, the ingot was removed from the mill and rolled, and the ingot was rough-rolled and finish-rolled to finally form a 15 mm thick steel plate, with the final rolling temperature controlled at 840 °C. The steel plate was air-cooled to room temperature after being cooled down to 420 °C by laminar flow cooling. A cross-sectional specimen (12 × 12 × 10 mm) was obtained from the rolled steel plate via EDM cutting; the sample was then ground and polished (200–1000 grit) before further treatment. After being sanded and polished by 200 to 1000 grit sandpaper, the samples were subjected to an etching treatment using a 4% nitric acid alcohol solution for about 12 s. Subsequently, the samples were alcohol-cleaned and blow-dried, and the basic tissue characteristics were observed using an optical microscope, Zeiss Axio Imager M2 (Carl Zeiss AG, Oberkochen, Germany), followed by a scanning electron microscope (Quten650 SEM, Thermo Fisher Scientific/FEI, Waltham, MA, USA) to further characterize the microstructural evolution of the test steel [20]. EDM was utilized to cut and process the required impact and tensile specimens, and the impact test was carried out in accordance with the GB/T229.1-2020 standard [21] test method, with the specimen specifications and dimensions as shown in Figure 1a, while the room temperature tensile test was carried out in accordance with the GB/T228.1-2021 standard method [22], with specimen specifications and dimensions as shown in Figure 1b.

**Figure 2:**
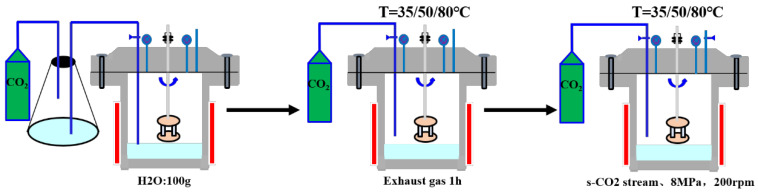
Supercritical CO_2_ corrosion experimental device diagram.

The corrosion tests focused on evaluating the influence of temperature and Cr content on corrosion behavior in a water-saturated CO_2_ environment. Four groups of test steels were exposed to corrosion conditions at 35 °C, 50 °C, and 80 °C for one week to elucidate the roles of temperature and Cr content in supercritical CO_2_ corrosion. Prior to testing, specimen surfaces were polished using 800-mesh sandpaper to ensure uniform roughness, followed by cleaning with anhydrous ethanol and drying with compressed air. The specimens were then weighed using an electronic balance with a precision of 0.01 mg. For consistency, a total of 12 corrosion tablets (with four parallel specimens per group) were installed simultaneously to ensure that all test steels experienced identical environmental conditions, thereby minimizing extraneous errors. The corrosion device is shown in Figure 2.

The specimens were mounted on a rotating cage and placed inside an autoclave, providing an exposed surface area of 500 mm^2^ to the supercritical CO_2_. Initially, 100 g of CO_2_-saturated water was added to the bottom of the autoclave, ensuring a water-saturated state as supercritical CO_2_ is always water-saturated [24]. Air was purged from the system using CO_2_ gas cylinders for 1 h, after which the autoclave outlet valve was closed. The autoclave was then set to the desired temperature, and the heating system activated. High-pressure CO_2_ was introduced into the autoclave via booster pumps until a pressure of 8 MPa was reached. Subsequently, all valves were closed, and the stirring mechanism was activated at a controlled speed of 200 rpm to simulate the dynamic conditions of supercritical CO_2_ pipeline transport. The autoclave was maintained at the set temperature and pressure for 168 h.

After the test period, the autoclave was opened and the corrosion specimens were retrieved. Some specimens were used for corrosion rate determination, while others underwent XRD analysis to characterize the corrosion products. Additional specimens were reserved for detailed analysis of the rust layer’s structure and morphology.

## 3. Results

### 3.1. Microstructure and Mechanical Properties

**Figure 3:**
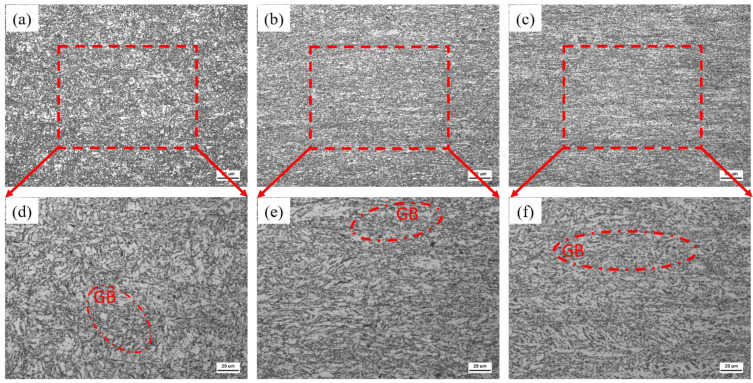
OM microstructure state of the experimental steels A (**a**,**d**), B (**b**,**e**), and C (**c**,**f**).

Optical microscopy and metallographic Figure 3 observations reveal that all three test steels exhibit microstructures predominantly composed of granular bainite (GB), with similar organizational types and grain sizes. Detailed analysis shows that, in addition to the dominant GB matrix, a minor martensite–austenite (MA) phase is present. Although the overall composite microstructures are consistent across the three steels, subtle variations in coarseness were noted—specifically, Steel C exhibits a slightly finer microstructure compared to Steels A and B. Overall, the microstructural characteristics of the three test steels are largely analogous.

**Figure 4:**
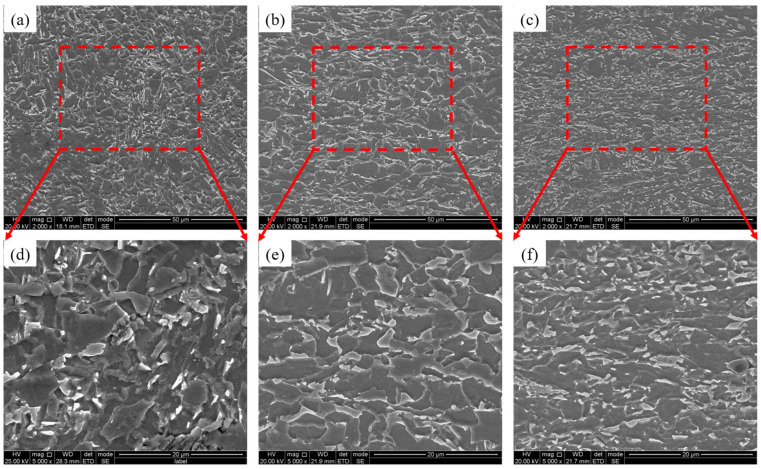
SEM microstructure state of the experimental steels A (**a**,**d**), B (**b**,**e**), and C (**c**,**f**).

Figure 4 illustrates the SEM microstructures of the three test steels. In Figure 4a,d, Steel A exhibits a matrix predominantly composed of granular bainite with a small number of diffusely distributed MA phase islands, indicating that its heat treatment produced a balanced and stable microstructure. In contrast, the microstructure of Steel B contains a significantly higher volume fraction of the MA phase, which is uniformly distributed; although the MA phase sizes are comparable to those in Steel A, the increased quantity suggests a greater potential for strengthening. Moreover, as shown in Figure 4f, Steel C is also dominated by granular bainite, but with a notably higher amount of MA phase that is considerably refined—exhibiting reduced particle size and a finer morphology. This suggests that Steel C may have undergone more extensive dynamic recrystallization and phase transformation during hot rolling, resulting in a finer and more homogeneously distributed MA phase, which is beneficial for enhancing material strength [25].

**Figure 5:**
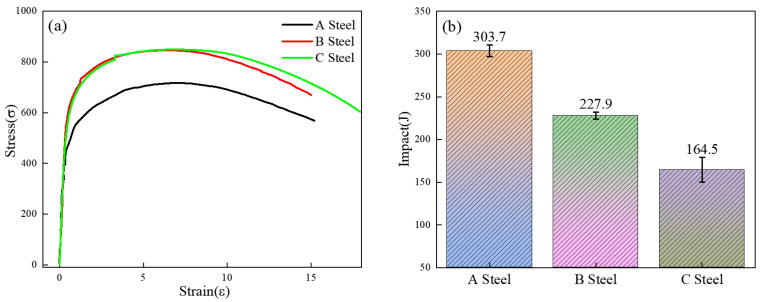
Stress–strain curve and low-temperature impact toughness of the three test steels. (**a**): Stress-strain curve, (**b**): Low-temperature impact of three experimental steels.

Figure 5a presents the stress–strain curves for the three test steels. It is evident that Test Steel A exhibits relatively low mechanical performance, with a tensile strength of approximately 650 MPa and a yield strength of around 490 MPa. In contrast, Test Steel B shows intermediate strength—with a tensile strength of about 786.5 MPa and a yield strength near 507 MPa—while Test Steel C exhibits the highest strength levels. Figure 5b illustrates that the impact toughness decreases as the strength increases, with impact work dropping from 303.7 J to 164.5 J. This trend is partly attributed to the progressive increase in Cr content among the steels, which enhances hardenability and leads to a higher proportion of the MA phase in the matrix. Additionally, the microstructure is refined, resulting in a significant reduction in MA phase particle size [26]. Overall, these observations suggest a close relationship between mechanical properties and microstructural evolution; specifically, the increase in strength accompanied by a decrease in toughness reflects changes in the morphology and size of the MA phase in the steels.

### 3.2. Weight Loss and Corrosion Rate

**Figure 6:**
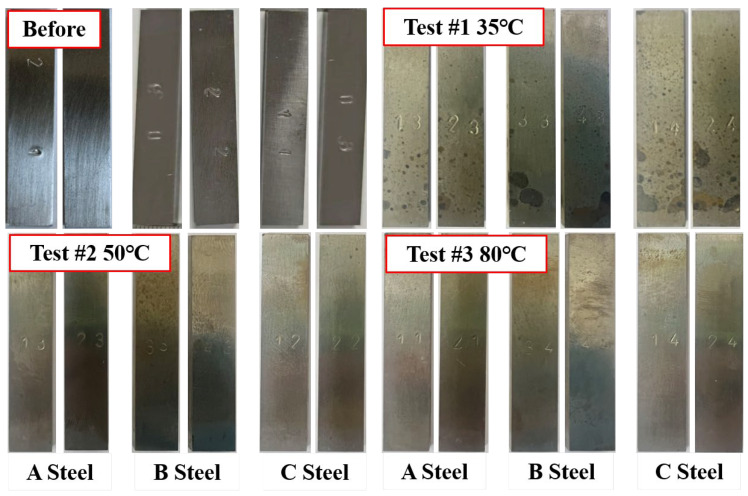
Photographic images before and after testing in H_2_O saturated S-CO_2_ at 8 MPa, at different temperatures.

Photographic images of the corroded specimens before and after tests #1–3 are presented in Figure 6. When exposed to water-saturated supercritical CO_2_ at three different temperatures, the steel surfaces generally exhibited a light brown hue with sporadic round black corrosion products, indicating a relatively mild corrosive environment. However, in Test #1 at 35 °C, numerous black, round corrosion pits were observed on the sample surface, underscoring the significant impact of temperature on the corrosion process. To accurately determine the corrosion rate, weight-loss measurements were conducted on four parallel samples under identical test conditions. The corrosion products were chemically removed using a pickling solution composed of 500 mL hydrochloric acid, 500 mL deionized water, and 3.5 g hexamethylene tetramine, following ASTM G1-03 [27]. Consequently, the corrosion rate (mm/y) of the steel specimens was calculated as follows:

(1) $$Corr\cdot Rat\mathrm{e}=\frac{8.76\times 10^{4}\times w}{s\rho t}$$

where w is the mass loss of the sample after corrosion products before and after the corrosion test; *s* denotes the area fraction of steel; ρ is the density of the experimental steel; and t is the corrosion test time (h).

**Figure 7:**
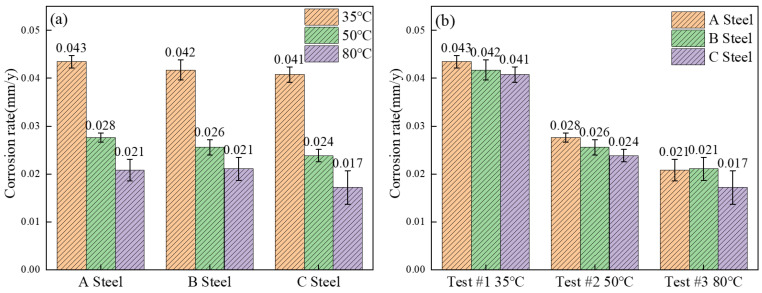
Corrosion rate of three test steels at 8MPa and different temperatures. (**a**) Corrosion rate of experimental steel at different temperatures, (**b**) Corrosion rate of different experimental steels at the same temperature.

Figure 7a illustrates that, in a water-saturated S-CO_2_ environment, the corrosion rates of the three test steels decrease with increasing temperature, with the maximum rate observed at 35 °C. Preliminary analysis suggests that this phenomenon may be attributed to both the thermodynamic and kinetic aspects of corrosion, as well as the protective effects of the corrosion product film. As temperature rises, the physicochemical properties of supercritical CO_2_ become more active, which accelerates the corrosion process [28]. However, as corrosion progresses, a protective film gradually forms on the steel surface, especially under relatively mild corrosive conditions. Once established, this film effectively impedes further corrosion by S-CO_2_, leading to a reduction in the overall corrosion rate [29].

Figure 7b further shows that the corrosion rate of the test steel decreases significantly with increasing Cr content. The interplay between Cr content, temperature, and corrosion is clarified by comparing Steels A and C: rust layer thickness and composition are contrasted across temperature conditions to reveal how temperature drives corrosion behavior. These findings offer critical data that support efforts to optimize the corrosion resistance of the test steels for practical applications.

### 3.3. Rust Layer Morphology and Composition

**Figure 8:**
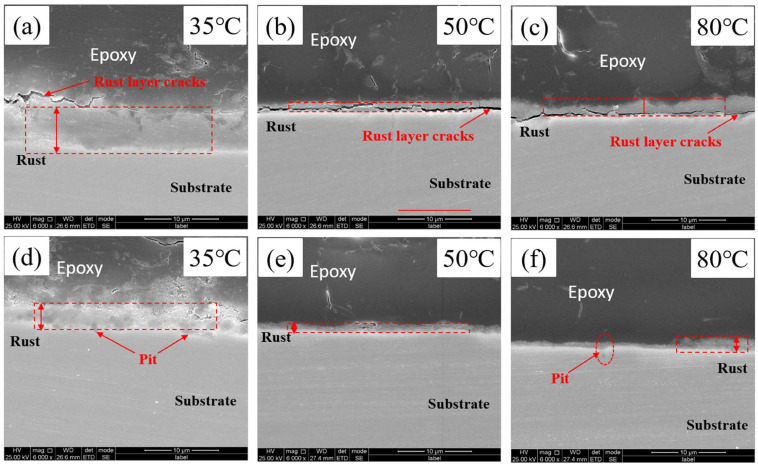
Cross-sectional morphology of the rust layer of the three experimental steels after corrosion: A steel: (**a**–**c**); C steel: (**d**–**f**).

Figure 8a–c illustrate the cross-sectional morphology of the rust layer on Test Steel A at different temperatures. Comparative analysis reveals that the rust layer of Steel A exhibits deeper cracks overall. Notably, at 50 °C and 80 °C, the rust layer thickness ranges from 1 to 3 μm, whereas at 35 °C, the rust layer is significantly thicker and uniformly distributed at approximately 5 to 7 μm. Moreover, at 35 °C, the rust layer not only becomes thicker but also shows a greater number of localized corrosion pits, indicating a more intense localized corrosion process. This phenomenon is tentatively attributed to the instability of the supercritical CO_2_ system at 35 °C and 8 MPa; under these conditions, fluctuations in the physicochemical properties may trigger the transition of CO_2_ from the supercritical to the gaseous state, causing the dissolved water phase to precipitate and form carbonic acid on the steel surface, thereby intensifying localized corrosion [30].

Figure 8d–f display the cross-sectional morphology of the rust layer on Test Steel C at various temperatures. In contrast to Steel A, Steel C exhibits a generally denser rust layer with fewer cracks. Although the rust layer thickness of Steel C is comparable to that of Steel A, its structure is noticeably more compact. This improvement in density is likely related to the participation of Cr in the corrosion process; the literature suggests that the hydrolysis of Cr into Cr_2_O_3_ in acidic environments contributes to stabilizing and enhancing the protective nature of the corrosion film [31].

To further elucidate the effects of temperature and Cr content on the corrosion behavior in water-saturated S-CO_2_, the morphology and elemental composition of the corrosion products on the rust layer surface were analyzed using scanning electron microscopy (SEM) and energy-dispersive spectroscopy (EDS).

**Figure 9:**
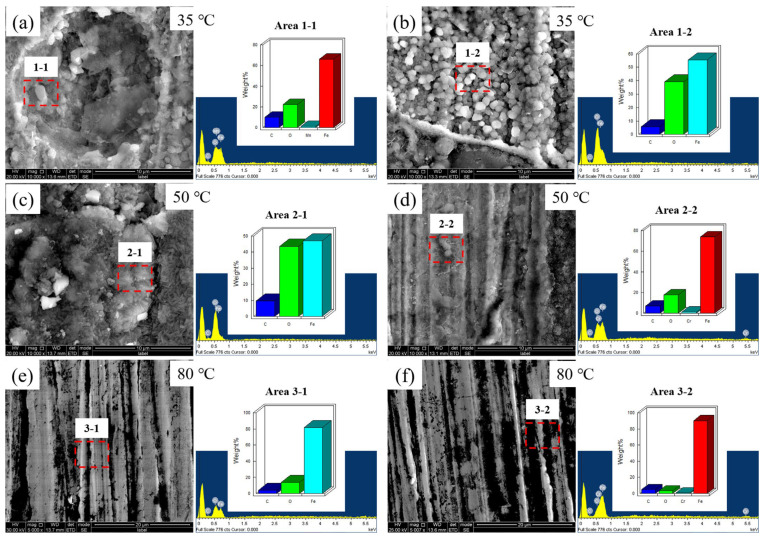
SEM and EDS results collected on test steel coupons after the exposure to 8 MPa H_2_O-saturated S-CO_2_ streams with different temperatures: A steel: (**a**,**c**,**e**); C steel: (**b**,**d**,**f**).

Figure 9a,c,e present the temperature-dependent corrosion morphology and corresponding EDS analysis of Test Steel A in H_2_O-saturated supercritical CO_2_ (S-CO_2_) environments. At 35 °C/8MPa (Figure 9a), the surface exhibits pronounced circular corrosion pits accompanied by bright particulate deposits within the rust layer. This localized corrosion pattern likely originates from phase instability during experimental cycling: mechanical agitation from rotational components combined with transient pressure–temperature fluctuations near the specimen surface may have induced aqueous phase separation from the S-CO_2_ medium. The resulting intermittent water droplet impingement creates aggressive microenvironments conducive to pit nucleation and propagation.

**Figure 10:**
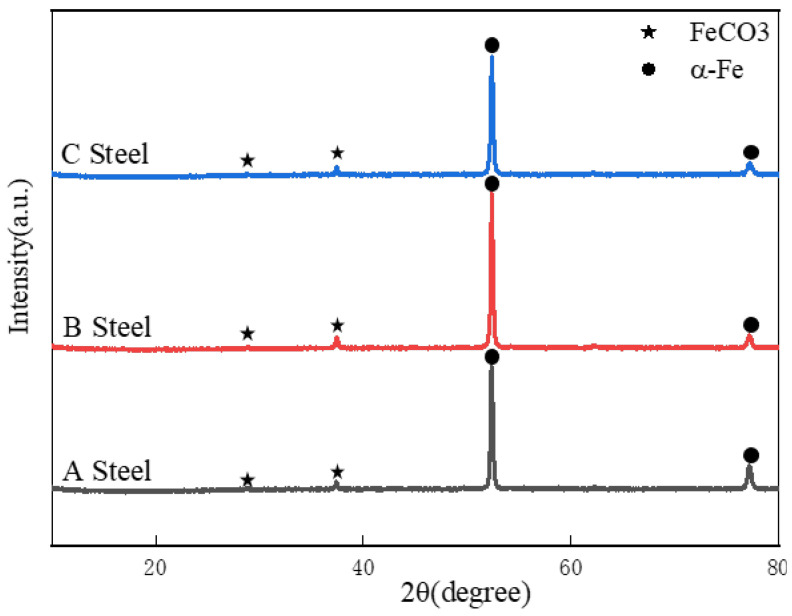
XRD of three experimental steels corroded by water-saturated supercritical CO_2_ for 168 h at 8 MPa and 50 °C.

In contrast, the 80 °C condition (Figure 9e) demonstrates markedly reduced corrosion severity, with specimens developing a continuous, dense FeCO_3_ product film (3–5 μm thickness), confirmed through combined EDS elemental mapping (C, O, Fe predominance) and XRD crystallographic analysis (Figure 10). This temperature-dependent morphological transition suggests enhanced passivation capability at elevated thermal conditions.

A parallel analysis of the control steel C (Figure 9b,d,f) reveals that the morphology of corrosion products appears superficially similar to that of steel A under identical conditions. Notably, while no Cr enrichment was detected in the rust layer of steel A, a localized Cr enrichment is evident in the rust layer of steel C. This contrast likely correlates with the Cr content of the steels, suggesting a potential chromium migration/redeposition mechanism within the supercritical CO_2_/H_2_O (S-CO_2_/H_2_O) system. Such a phenomenon necessitates further investigation into the interfacial reaction kinetics, as comprehensively discussed in Section 4.

## 4. Discussion

### Fine Analysis of MA Microstructure

**Figure 11:**
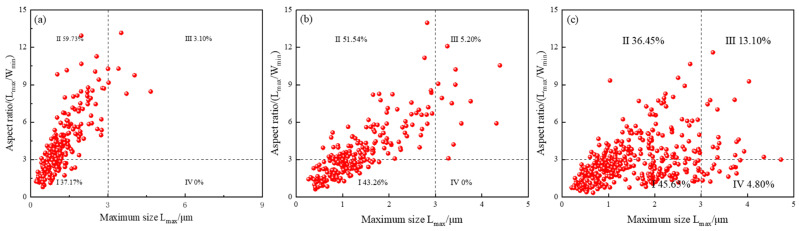
Size distribution of MA group elements of three experimental steels; (**a**) A steel; (**b**) B steel; (**c**) C steel.

In this study, the dual effects of chromium addition (0.38–1.17wt.%) on martensite–austenite (MA) constituents were systematically investigated in bainitic steels. Image-Pro was used to quantitatively analyze 10 random 1000× scanning electron microscopy (SEM) fields (area threshold > 0.1 μm^2^) to obtain Figure 11, the size distribution of MA components. Revealed that increasing Cr content not only refined the bainitic microstructure but also paradoxically increased MA area fraction from 2.4% ± 0.3% (0.38%Cr) to 7.2% ± 0.5% (1.17%Cr), while reducing equivalent diameter from 1.8 ± 0.2 μm to 1.2 ± 0.1 μm. This inverse size–content relationship is attributed to Cr’s dual roles: (1) solute drag effect via interfacial Cr segregation, which restricts MA growth by impeding austenite/ferrite boundary mobility, and (2) accelerated bainitic ferrite nucleation under higher Cr levels, confining residual austenite into finer inter-lath domains. The refined MA architecture enhances corrosion resistance through two synergistic mechanisms—submicron MA particles mitigate passive film (Cr(OH)_3_/Cr_2_O_3_) stress concentration to inhibit cracking, while their increased dispersion density (4.1% area fraction) at prior austenite grain boundaries obstructs CO_3_^2−^ diffusion, collectively improving pitting resistance and film stability in aggressive CO_2_ environments.

This investigation reveals a distinctive inverse correlation between temperature and corrosion rate in H_2_O-saturated supercritical CO_2_ (S-CO_2_) environments, with minimum degradation observed at 80 °C (Figure 7). This non-monotonic thermal dependence suggests competing temperature-activated mechanisms governing corrosion processes in S-CO_2_ systems—a phenomenon requiring careful decoupling of thermodynamic driving forces and kinetic barriers. Notably, the findings contrast with established patterns in undersaturated systems: Hua et al. [16] documented increasing corrosion rates with decreasing temperature in water-unsaturated S-CO_2_ environments, while Choi et al. [32] reported similar thermal dependence in H_2_S-contaminated S-CO_2_ systems at 12 MPa, observing order-of-magnitude corrosion acceleration at lower temperatures. These contradictory observations underscore water saturation’s pivotal role in modulating corrosion mechanisms—potentially altering the rate-determining steps from surface reaction control to mass transport limitation.

The complexity of thermal effects is further exemplified by Sui et al.’s non-monotonic corrosion profile for X65 steel in H_2_S-containing S-CO_2_ at 8 MPa, where corrosion rates initially increased before declining with rising temperature. Corrosion behavior in water-saturated supercritical CO_2_ environments without impurities exhibits a consistent reduction in corrosion rates across a temperature range of 35–80 °C. This trend suggests a shift in the dominant corrosion mechanisms as temperature increases.

To enhance clarity and precision, the sentence can be revised as follows:

Thermodynamic Analysis: Utilizing the Spycher-Reed (2003) [33] CO_2_-H_2_O mutual solubility model (Table 2), calculate phase-specific corrosivity indices. The experimental conditions yield CO_2_ densities of 450–650 kg/m^3^, with aqueous phase fractions below 2.5 vol%, indicating the following:

1. Reduced H_2_O activity in S-CO_2_ phase at elevated temperatures;
2. Diminished electrochemical reaction driving forces at higher thermal energy states.

**Table 2:** Mutual solubility of water and carbon dioxide in supercritical phase.

Temperature (°C)	Pressure (bar)	CO_2_ Density (kg/m^3^)	CO_2_ in Water (ppm)	Water in CO_2_ (ppm)
35	80	489.818	2.231 × 10^4^	3.381 × 10^3^
50	80	219.217	1.757 × 10^4^	3.197 × 10^3^
80	80	186.314	1.329 × 10^4^	1.196 × 10^4^

This systematic separation of thermodynamic favorability and kinetic accessibility provides a mechanistic foundation for interpreting the observed thermal trends. Table 2 shows the density of CO_2_ and the mutual solubility with water under the test conditions of this study:

(2) $$x_{CO_{2}}=\frac{\emptyset_{CO_{2}}(1-y_{H_{2}O})P_{tot}}{55.508K_{CO_{2}(g)}^{0}}exp(-\frac{(p-p^{0})\bar{V_{CO_{2}}}}{RT})$$

(3) $$y_{H_{2}O}=\frac{K_{H_{2}O}^{0}(1-x_{CO_{2}})}{\emptyset_{H_{2}O}P_{tot}}exp(\frac{(p-p^{0})\bar{V_{H_{2}O}}}{RT})$$

where in the above equation, *ɸCO*_2_ is the CO_2_ fugacity coefficient; *Ptot* is the total pressure; *K^0^* is the equilibrium constant; P is the partial pressure (MPa); *P0* is the reference pressure; VCO_2_ is the average fractional molar volume of carbon dioxide (cm^3^/mol); *R* is the gas constant(8.314 J/mol·K); T is the temperature (K); and similarly, VH_2_O is the average fractional molar volume of pure water; and *ɸH_2_O* is theH_2_O fugacity coefficient (all these constants can be obtained from the literature).

- CO_2_ density dependence: 1: Density decreases exponentially from 489.82 kg/m^3^ at 35 °C (308.15 K) to 186.31 kg/m^3^ at 80 °C (353.15 K) under constant pressure (8 MPa); 2: Corresponds to a 62% reduction in CO_2_ phase density across the tested thermal range;
- Solubility behavior: 1: H_2_O solubility in CO_2_-rich phase: 3381 ppm at 35 °C vs. 3197 ppm at 50 °C (Δ = 5.4%); 2: CO_2_ solubility in aqueous phase decreases monotonically with temperature;
- Mass transport implications: 1: Despite similar molar solubilities at 35 °C and 50 °C, the volumetric H_2_O concentration in the CO_2_ phase exhibits strong temperature dependence; 2: Demonstrates a 38.4% reduction in dissolved H_2_O mass per unit volume despite only a 5.4% decrease in molar fraction.

Mechanistic interpretation: This inverse proportionality between CO_2_ density and dissolved H_2_O mass concentration provides critical insight into the observed temperature-dependent corrosion behavior.

At lower temperatures (35–50 °C): 1. Higher CO_2_ phase density enables greater H_2_O mass retention per unit volume; 2. Creates localized aqueous microenvironments conducive to electrochemical corrosion.

At elevated temperatures (>60 °C): 1. Reduced CO_2_ density limits H_2_O dissolution capacity; 2. Promotes the formation of protective FeCO_3_ films through decreased water availability

**Figure 12:**
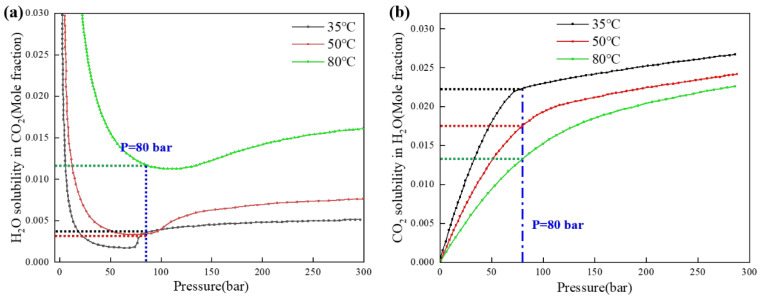
Solubility curves with varying temperature and pressure; (**a**) water solubility in CO_2_; (**b**) CO_2_ solubility in water.

Theoretical–practical correlation:

The computational results align with experimental corrosion rate measurements (Figure 12), where the 1.6× higher H_2_O mass concentration at 35 °C correlates with 2.3× greater corrosion rates compared to 50 °C. This quantitative relationship confirms that phase-stability-governed H_2_O distribution fundamentally controls corrosion mechanisms in S-CO_2_ systems.

Corrosion within the supercritical CO_2_ autoclave is categorized into two primary mechanisms, which are considered to be competitive. One is the dissolution of water in the supercritical CO_2_ fluid, whereby the fluid is corrosive and comes into contact with the steel surface, thereby causing corrosion [34,35]. The mechanism is as follows: molecular water dissolved in the S-CO_2_ phase (≤2.5 vol%) interacts with steel surfaces through the following:

- Electrochemical dissolution at CO_2_/Fe interfaces;
- Carbonic acid (H_2_CO_3_) formation via CO_2_ hydration;
- Dominance conditions: Prevails at higher S-CO_2_ densities (>400 kg/m^3^), where dissolved H_2_O mass concentration peaks.

The other is that the aqueous phase precipitates out of the supercritical CO_2_ fluid to form condensate, and the CO_2_ dissolves into the condensate to form an acidic solution that causes corrosion [36,37]. The mechanism is as follows: Phase-separated H_2_O droplets (formed through S-CO_2_ instability) absorb CO_2_ to create carbonic acid solutions:

- Local pH reduction to 3.2–4.1 (calculated using HSC Chemistry);
- Microenvironmental galvanic cell formation;
- Dominance conditions: Favored near critical point (30–35 °C/8 MPa), where minor T/P fluctuations trigger phase separation.

As the temperature increases (35 °C, 50 °C, 80 °C), the solubility of water in supercritical CO_2_ decreases and then increases, which affects the composition of the corrosive medium, and the supercritical CO_2_ fluid corrosivity shows a tendency of decreasing and then increasing. From one point of view, with the increase in temperature (35 °C, 50 °C, 80 °C), the solubility of CO_2_ in H_2_O is decreasing, which means that the acidity of the solution is weakened, and the corrosiveness of the aqueous phase is also weakened. Combined with Figure 6 and Table 2, more localized corrosion occurs on the surface of the 35 °C test steel, also due to the environmental conditions of 35 °C and 8MPa, which are close to the critical zone of supercritical CO_2_, at which time the supercritical state of CO_2_ is unstable, and weak changes in temperature and pressure may lead to a CO_2_ phase change, which can cause a drastic decrease in the solubility of the aqueous phase, leading to more serious localized corrosion.

35 °C (Near-Critical Regime): 1. CO_2_ density: 489.82 kg/m^3^ (Table 2); 2. High H_2_O dissolution capacity (1.657 kg/m^3^) enables Type I dominance; 3. Metastable S-CO_2_phase → Frequent phase separation → Type II localized attack; 4. Synergistic effects yield maximum corrosion rates (0.38 mm/y).

50 °C (Stable S-CO_2_): 2. Density drops to 319.45 kg/m^3^ (−34.8%); 2. Reduced H_2_O dissolution (1.021 kg/m^3^) weakens Type I; 3. Stable single-phase S-CO_2_ suppresses Type II; 4. Corrosion rate decreases to 0.28 mm/y.

80 °C (High-Temperature S-CO_2_): 1. Density plummets to 186.31 kg/m^3^ (−62%); 2. Minimal H_2_O dissolution (0.512 kg/m^3^) eliminates Type I; 3. Thermodynamically favored FeCO3 passivation dominates; 4. Corrosion rate minimizes at 0.18 mm/y.

Competitive mechanism analysis:

The observed inverse temperature–corrosion rate relationship arises from two superimposed effects: Type I Suppression: Reduced H_2_O mass concentration in S-CO_2_phase with rising temperature (Figure 11a. Type II Elimination: Stabilized single-phase S-CO_2_ eliminating droplet formation (Figure 11b).

The superiority of the corrosion resistance of test steels also involves a competitive relationship between the occurrence of corrosion and the overall protection of the rust layer. On the one hand, as the temperature increases, molecules have more energy and the number of molecular collisions with energy in excess of the activation energy increases, leading to an accelerated corrosion rate [38]. On the other hand, as corrosion behavior occurs, corrosion products are subsequently precipitated, and the gradual formation of a corrosion product film on the surface of the test steel will prevent further corrosion from occurring. The Cr effect is less pronounced at 35 °C and we discuss why (e.g., CO_2_ solubility/film breakdown more strongly drive the rate under those conditions).

The corrosion resistance of test steels in supercritical CO_2_ (S-CO_2_) environments emerges from a kinetic–thermodynamic competition between two temperature-activated processes:

1. Corrosion Acceleration Mechanism (Arrhenius Dominance):

■ Thermal energy provision lowers activation barriers for:
  ◆ Charge transfer reactions: $E_{a}^{CT}\propto T^{-1}$;
  ◆ Ionic transport through double layers;
■ Increased frequency of energy-sufficient molecular collisions (Boltzmann distribution):
  (4) $$K_{corr}\propto exp(-\frac{E_{a}}{RT})$$
■ Predicts +2.3× corrosion rate increase per 25 °C rise (35→60 °C) based on typical Ea ≈ 45 kJ/mol [38].

2. Passivation kinetics (nucleation-growth control):

■ Temperature-enhanced FeCO_3_ precipitation thermodynamics;
  ◆ Nucleation rate $J\propto exp(-\frac{16\pi r^{3}}{3k_{B}^{3}T^{3}(\ln S{)}^{2}})$ (Classical nucleation theory);
  ◆ Growth dominated by Fickian transport of Fe^2+^/CO_3_^2−^;
■ Above 60 °C, achieves critical nuclei density ($N_{c}>10^{11}{cm}^{-3}$) for continuous film formation [39].

This inversion of thermal dependence above 50 °C demonstrates a critical transition from a reaction-controlled to a diffusion–passivation dominated regime. In contrast with previous works (e.g., Xiang et al. 2013) [40], which reported decreasing corrosion rates with increasing temperature in water-saturated S-CO_2_ for carbon steels and corrosion-resistant alloys, the present results not only confirm similar temperature trends but also demonstrate sharper rate reductions attributable to Cr content combined with microstructural refinement and protective film formation. Compared to Xu et al. [41], whose experiments in unsaturated CO_2_ showed dramatic rate increases with higher water content, the saturated, impurity-free conditions used here lead to nearly monotonic rate decrease from 35 to 80 °C. Furthermore, while AMPP 2022 Firouzdor, V. [42] found Cr content differences to be modest at lower temperatures under impurity-containing environments, this study shows that under clean, saturated CO_2_, Cr’s effect becomes more pronounced as temperature increases and protective films mature.

Under the experimental conditions, corrosion within the supercritical CO_2_ autoclave is categorized into two primary mechanisms, which are considered to be competitive. One is dominated by supercritical CO_2_ fluid; for the chemical reaction, mainly the acidic liquid and steel surface direct the redox reaction; there may be a weak charge transfer, but in the corrosion process it is not dominant. The reaction equation is as follows:

(5) $$CO_{2}+H_{2}O\to H_{2}CO_{3}\surd$$

(6) $$Fe+H_{2}CO_{3}\to FeCO_{3}+H_{2}\surd$$

(7) $$Cr+H_{2}CO_{3}\to Cr_{2}(CO_{3}{)}_{3}+H_{2}\surd$$

In the supercritical CO_2_ environment, Cr_2_(CO_3_)_3_ is less stable, and should further hydrolysis occur, this will generate Cr(OH)_3_. Due to the acidic environment, Cr(OH)_3_ is not stable and very easy to hydrolyze, generating Cr_2_O_3_ stable corrosion products. The reaction equation is as follows:

(8) $$Cr_{2}(CO_{3}{)}_{3}+3H_{2}O\to 2Cr(OH{)}_{3}+3CO_{2}\surd$$

(9) $$2Cr(OH{)}_{3}\to Cr_{2}O_{3}+3H_{2}O\surd$$

**Figure 13:**
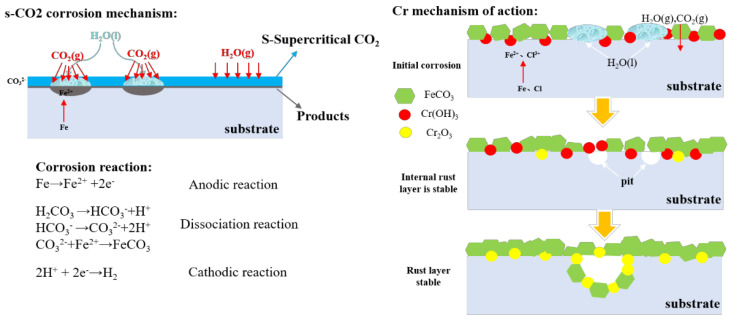
Competitive mechanisms for aqueous and supercritical CO_2_ corrosion.

The other is aqueous-phase-dominated electrochemical generation, which corresponds to the localized corrosion during supercritical CO_2_ corrosion. The corrosion process is mainly controlled by charge transfer; this corrosion principle has been analyzed in detail in previous studies [43] and will not be repeated here. Figure 13 is a schematic diagram of the influence mechanism of temperature and Cr content.

Several limitations should be acknowledged. First, the experiments were conducted in pure, impurity-free CO_2_; real, industrial CO_2_ often contains gases such as H_2_S, O_2_, and SO_2_, which can significantly alter corrosion kinetics and film chemistry. Second, the temperature range investigated (35–80 °C) may not cover extreme service conditions (lower or higher) encountered in some pipeline systems. Third, only chromium (Cr) content was varied; other alloying elements (e.g., Mo, Ni) and microstructural variables may also impact corrosion behavior. Finally, sampling and image analysis (SEM fields) may not fully capture microscale heterogeneity across an entire component. These limitations recommend caution when generalizing the results to complex or impurity-rich environments and motivate future work.

## 5. Conclusions

This study systematically investigates the coupled effects of chromium alloying and temperature conditions on corrosion mechanisms in water-saturated supercritical CO_2_ (S-CO_2_) environments, providing critical insights for pipeline material selection and transportation protocol optimization. The principal findings are summarized as follows:

(1) With the increase in Cr content, the basic type of the three test steels is granular bainite, but the microstructure is refined (25% reduction in average packet size). The MA group element content in particular increases (8.2% → 14.7%), but the size is refined. The strength of the test steel increases and the toughness decreases.
(2) In the water-saturated supercritical CO_2_ environment, with the increase in Cr content, the corrosion rate shows a decreasing trend (0.38Cr:0.0379 mm/y→1.17Cr:0.0291 mm/y). The Cr element is involved in the supercritical CO_2_ reaction. Carbonic acid combined with hydration, and ultimately the formation of stable Cr_2_O_3_, improves the densification and stability of the rust layer.
(3) In the water-saturated supercritical CO_2_ environment, with the increase in ambient temperature, the corrosion rate shows a decreasing trend; at 35 °C, the corrosion rate reaches its highest value. At 35 °C, the environmental state close to the critical point of CO_2_, the water phase is easy to precipitate from the supercritical CO_2_, and the supercritical CO_2_ density is at the maximum, 489.818 kg/m^3^. At this time, the CO_2_ has maximum solubility in the H_2_O phase, and corrosion also occurs in the form of H_2_O. The corrosion is dominated by the electrochemical corrosion of the aqueous phase. With the further increase in temperature, corrosion is dominated by chemical corrosion, and with the increase in temperature, promoting the formation and stabilization of corrosion film is conducive to further reducing the corrosion rate.
(4) In the water-saturated supercritical CO_2_ environment, corrosion is mainly divided into two types, the first being water dissolved into the supercritical CO_2_ to form carbonic acid, which directly corrodes the surface of the experimental steel; this corrosion is generally uniform corrosion. This corrosion is mainly dependent on the amount of water dissolved; the corrosion is weak. The other is direct contact between the water phase from the supercritical CO_2_ precipitation and the steel surface, generally resulting in localized corrosion; this is the corrosion principle for electrochemical corrosion, and the steel is more affected.

## Figures and Tables

**Table 1:** Chemical compositions of the steels (wt.%).

Steels	C	Si	Mn	Cr	Ni+Mo	Cu	Nb	Ti	Al
A	0.050	0.24	1.39	0.38	0.4	0.16	0.042	0.016	0.031
B	0.055	0.25	1.38	0.75	0.4	0.16	0.041	0.015	0.033
C	0.055	0.25	1.38	1.17	0.4	0.16	0.041	0.015	0.033

## Data Availability

The original contributions presented in this study are included in the article. Further inquiries can be directed to the corresponding author.
